# A235 “WHAT’S SPRUE WITH YOU?”: OLMESARTAN-INDUCED ENTEROPATHY AS AN UNCOMMON MIMICKER OF CELIAC DISEASE

**DOI:** 10.1093/jcag/gwac036.235

**Published:** 2023-03-07

**Authors:** F Milne, C E Orr, J Flemming

**Affiliations:** Queen's University, Kingston, Canada

## Abstract

**Background:**

Olmesartan-induced enteropathy is an uncommon mimicker of celiac disease. Symptoms include diarrhea and weight loss and pathology shows villous atrophy, intraepithelial lymphocytes, and subepithelial collagen deposition. In contrast to celiac disease, tissue transglutaminase (TTG) is not elevated and gluten avoidance does not induce clinical or histologic improvement. Both clinical and histologic findings are reversible with cessation of pharmacologic therapy.

**Purpose:**

To raise clinician awareness of an uncommon but reversible cause of enteropathy.

**Method:**

A 67-year old man presented with 3 months of progressive diarrhea and 35lb weight loss on a background of hypertension, dyslipidemia, and type 2 diabetes. His medications included olmesartan, amlodipine, metformin, aspirin, and atorvastatin, none of which were new. Infectious stool studies, serum and fecal inflammatory markers, imaging, TTG and IgA were normal.

Panendoscopy revealed duodenitis with biopsies consistent with active enteritis, intraepithelial lymphocytosis and villous shortening suggestive of olmesartan-induced enteropathy given negative TTG. Olmesartan was discontinued.

**Result(s):**

His symptoms completely resolved over several weeks after discontinuation.

**Conclusion(s):**

Olmesartan is an uncommon but reversible mimicker of celiac disease. It was first described in 2012 in a case series of 22 patients, all of whom had negative celiac serology and demonstrated histologic and clinical improvement with cessation of olmesartan. Time from medication initiation to onset of symptoms ranged from 6 months to 7 years, with a mean of 3.1 years.

While case reports exist of angiotensin-receptor blockers as causative agents, the most common is olmesartan, implicated in 94% of ARB-induced enteropathy cases in a recent systematic review.

The exact mechanism of injury is unknown. The delay in onset of symptoms suggests a cell-mediated immunity phenomenon. An immune-mediated reaction is also supported by the high prevalence of HLA-DQ2/DQ8 haplotypes (71%) among affected individuals vs. 30-40% in the general population. Olmesartan may be implicated more strongly than other ARBs due to its higher affinity for AT1 receptors; theoretically leaving unsaturated AT2 receptors to bind angiotensin. AT2 receptors are known to induce cellular apoptosis, and unopposed cellular death could theoretically lead to villous atrophy.

The overall risk of developing enteropathy on olmesartan is rare, but not well quantified. This is likely due to both underrecognition, and variability in study designs leading to inability to perform meta-analysis on currently available data. Data from a French cohort estimates a crude incidence rate of 5.6 per 100,000 person years for patients on olmesartan developing intestinal malabsorption requiring hospitalization.

Despite its rarity, prescribers should be cognizant of this side effect which can evolve even after years of medical therapy.

**Please acknowledge all funding agencies by checking the applicable boxes below:**

None

**Disclosure of Interest:**

None Declared

